# Identification of the genetic basis of sow pelvic organ prolapse

**DOI:** 10.3389/fgene.2023.1154713

**Published:** 2023-04-18

**Authors:** Vishesh Bhatia, Tomas Stevens, Martijn F. L. Derks, Jenelle Dunkelberger, Egbert F. Knol, Jason W. Ross, Jack C. M. Dekkers

**Affiliations:** ^1^ Department of Animal Science, Iowa State University, Ames, IA, United States; ^2^ Topigs Norsvin Research Center, Beuningen, Netherlands; ^3^ Topigs Norsvin, Burnsville, MN, United States

**Keywords:** prolapse, sow, heritability, genetics, GWAS, swine

## Abstract

**Introduction:** Pelvic organ prolapse (POP) is one contributor to recent increases in sow mortality that have been observed in some populations and environments, leading to financial losses and welfare concerns.

**Methods:** With inconsistent previous reports, the objective here was to investigate the role of genetics on susceptibility to POP, using data on 30,429 purebred sows, of which 14,186 were genotyped (25K), collected from 2012 to 2022 in two US multiplier farms with a high POP incidence of 7.1% among culled and dead sows and ranging from 2% to 4% of all sows present by parity. Given the low incidence of POP for parities 1 and >6, only data from parities 2 to 6 were retained for analyses. Genetic analyses were conducted both across parities, using cull data (culled for POP versus another reason), and by parity, using farrowing data. (culled for POP versus culled for another reason or not culled).

**Results and Discussion:** Estimates of heritability from univariate logit models on the underlying scale were 0.35 ± 0.02 for the across-parity analysis and ranged from 0.41 ± 0.03 in parity 2 to 0.15 ± 0.07 in parity 6 for the by-parity analyses. Estimates of genetic correlations of POP between parities based on bivariate linear models indicated a similar genetic basis of POP across parities but less similar with increasing distance between parities. Genome wide association analyses revealed six 1 Mb windows that explained more than 1% of the genetic variance in the across-parity data. Most regions were confirmed in several by-parity analyses. Functional analyses of the identified genomic regions showed a potential role of several genes on chromosomes 1, 3, 7, 10, 12, and 14 in susceptibility to POP, including the Estrogen Receptor gene. Gene set enrichment analyses showed that genomic regions that explained more variation for POP were enriched for several terms from custom transcriptome and gene ontology libraries.

**Conclusion:** The influence of genetics on susceptibility to POP in this population and environment was confirmed and several candidate genes and biological processes were identified that can be targeted to better understand and mitigate the incidence of POP.

## 1 Introduction

Pork production has seen an overall increase worldwide and is expected to continue to grow as the global human population reaches new milestones (Livestock and Poultry: World Markets and Trade | USDA Foreign Agricultural Service, 2022). To meet such growing pork demands, individually owned hog farms have transformed into commercially contracted swine production units with improved technologies and greater farm sizes. Most of these swine production units have common genetic suppliers, but they are scattered globally, leading to genetically similar pigs housed under different environmental conditions (Davis et al., 2022). Improvements in management and genetics have enabled a significant improvement in the production efficiency of sows through increases in the number of piglets born alive and weaned and consistent litter performance across parities. Unfortunately, a concomitant steady rise in sow mortality has adversely affected the global swine industry (Eckberg, 2022), leading to large financial losses, not only due to forced removal of sows from the production system, but also by losing their current and future litters. In the U.S. swine industry, major reasons for sow mortality include unknown/sudden death, feet/leg structure, and pelvic organ prolapse (POP) (Supakorn et al., 2017; Ross, 2019). In recent years, removal due to POP has been on a rise and has gained substantial attention as a significant welfare and production issue (Mahan-Riggs et al., 2016; Pittman, 2017; Supakorn et al., 2017). In Canada, a study reported 6.6% of deaths due to uterine prolapse in 1991 (Chagnon et al., 1991). Several reports from Europe have also identified removal of sows due to POP as a large hurdle for swine producers (Engblom et al., 2007; Iida et al., 2019). In regions of South America, POP has also been identified as a major reason of sow removal (Schwertz et al., 2021; Monteiro et al., 2022). Therefore, getting a better understanding of factors that affect incidence of POP across the globe has become of increasing importance.

Pelvic organ prolapse is characterized by the loss of support from tissues and muscles of the pelvic floor, leading to a drop of pelvic organs from their normal position (Jelovsek et al., 2007). Commonly defined as an anatomical disorder, POP is mostly diagnosed by the protruding of pelvic organs, including the vagina, rectum, urethra, bladder, uterus, or cervix. Vaginal, rectal, and uterine prolapse are the most common types found in sows, while rectal prolapse can occur in combination with uterine and vaginal prolapse (Supakorn et al., 2017). A steady increase in the incidence of POP over the years has been of major concern for swine farmers and breeders. The increase in incidence is persistent, even though there have been substantial reproductive improvements in the swine industry. In contrast, the incidence of POP has been relatively low in other livestock species, ranging from under 1% for cattle (Jackson et al., 2014) to between 1% and 6% for sheep (Carluccio et al., 2020). Depending on the severity of the prolapse, the opportunities for treatment of prolapse are limited and, therefore, prolapse typically leads to removal of the sow. If the condition is detected at an early stage and is less severe, the prolapse can be reversed using moderate force (Borobia-Belsué, 2006). Surgical repairs using the Buhner and purse-string suture have also been successful in treating prolapse (Anderson and Mulon, 2019). Amputation of the affected region is reserved as a last resort.

In pigs, several studies and mitigation strategies have been put forward to develop a better understanding of POP (Pittman, 2017). A major industry-wide survey of U.S. production systems identified several factors that were associated with incidence of POP in sows, suggesting that the causes of POP are multifactorial (Ross, 2019). These contributing factors ranged from body condition of the sow to management changes that either may be the sole culprit, or multiple factors that may interact, leading to higher risk of POP. A similar study in Spain identified being in the 16th week after service, being in parity 3 or higher, re-service, servicing in summer, autumn, or winter, shorter gestational length, fewer piglets born, and more stillborn piglets as factors that significantly increased the risk of prolapse (Iida et al., 2019). Efforts to better understand the physiological and endocrinological basis of the risk of POP have focused on understanding changes in endocrine signals (Kiefer et al., 2021c) and in the vaginal microbiota (Kiefer et al., 2021a; 2021b) during late gestation.

Genetics has been hypothesized to be another contributing factor to susceptibility to POP, but the few studies conducted have presented conflicting results. One study reported estimates of heritability for combined vaginal, rectal, and uterine prolapse of 0.03 ± 0.01 and 0.003 ± 0.003 based on linear and threshold models, respectively, indicating no genetic basis for susceptibility to POP (Supakorn et al., 2019). However, using data on purebred sows from a commercial maternal line from two US herds with high incidence of POP, Stevens et al. (2021) and Dunkelberger et al. (2022) reported threshold model heritability estimates of 0.22 for culling for POP (defined as vaginal or uterine prolapse) *versus* other reasons. The latter studies, however, used partially incomplete pedigrees because of some use of pooled semen. Therefore, the purpose of this study was to use genomic data to confirm the role of genetics in susceptibility to POP in sows in the data analysed by Dunkelberger et al. (2022) and to identify genomic regions and candidate genes that are associated with susceptibility to POP.

## 2 Materials and methods

### 2.1 Animals and data

All data included in the study were collected on purebred females from a Topigs Norsvin commercial line on two multiplier farms located in the Midwest of the U.S. These two farms were not located in close vicinity to each other and had different management. Farrowing and cull records for a total of 30,429 sows from 16 July 2012 to 31 May 2022 were included, of which 26,620 sows were culled or died during that period. Farrowing data included insemination and farrowing records for each parity of the sow, while the cull data included the date and parity when the sow was removed from the herd and the primary removal reason, including culling for POP, which combined vaginal prolapse and uterine prolapse, as clear differentiation of vaginal *versus* uterine prolapse by farm labor was unreliable. Given the low incidence of POP in parities 1 and 7 and higher, only data from parities two to six were retained for analysis. After quality control checks based on missing mortality dates, overdue sows (>116 days from insemination to farrowing), and inaccurate pedigree information, data on 20,094 sows remained, of which 17,700 were culled during the evaluated period. Of the cull records that remained after quality control, 442 sows had missing sire information due to use of pooled semen for insemination. These sows were, however not removed from the dataset. Of the 20,094 sows, 14,186 sows were genotyped (12,757 of the culled sows) using a 25K SNP panel and were used for downstream analysis. All animals were imputed up to a 50K panel using FImpute (Sargolzaei et al., 2014), resulting in genotype information for 48,075 markers.

Using these data, two types of analyses were conducted: by parity and across parities. For the across-parity analysis, only cull records were used, and the binary trait was set to 1 for sows that were culled due to POP and to 0 for sows that were removed for other reasons. For the by-parity analyses, the binary trait was set to 1 when the sow was removed in that parity due to POP and to 0 for sows that were either not removed during that parity or culled for another reason. Note that the POP phenotype data for each parity are independent, apart from the overlap of sows that farrowed in multiple parities.

### 2.2 Estimation of genetic parameters

The following univariate logistic regression animal model was used to estimate heritabilities of the by-parity and across-parity POP phenotypes, using AsReml 4.1 (Gilmour et al., 2015):

$$Logit\left(y\right)=Xb+Za+e$$

where *Logit(* ***y*** *)* is the vector of logits of the binary POP phenotypes, either by parity or across parities, ***b*** is the vector of fixed effects, ***a*** is the vector of random additive genetic effects, ***e*** is a vector of random residuals, and ***X*** and ***Z*** are incidence matrices relating *Logit(* ***y*** *)* to ***b*** and ***a***, respectively. The fixed effect consisted of the combination of herd, year, and quarter (HYQ) of the year of insemination. For the across-parity analysis, the latter referred to the quarter of insemination for the removal parity of the sow and removal parity was added as a fixed effect. In preliminary analyses, total number born was found to be significant (*p* < 0.001) as a covariate, with an estimated effect of −0.09, but 40% of the sows were culled before farrowing and, therefore, had no litter size information for that parity. Because of this, and to guard against removing genetic variation in susceptibility for POP, total number born was not included in the final model. Genetic relationships were either based on pedigree or genomic relationships. The latter were computed based on the SNP genotypes using calc_grm (Calus and Vandenplas, 2013). Estimates of heritability were obtained as the estimate of the additive genetic variance divided by the sum of the estimates of additive genetic and residual variance (Falconer and Mackay, 1996).

To determine whether the genetic control of susceptibility to POP was consistent across parities, bivariate models were fitted to the by-parity data to estimate the genetic correlations of susceptibility to POP between parities. Because bivariate threshold and logistic regression models failed to converge, a bivariate linear mixed model was used for this purpose, noting that linear and threshold models have been shown to yield similar estimates of genetic correlations (Mäntysaari et al., 1991; Elsaid and Elsayed, 2007). To allow statistical inferences, bivariate linear marker-effects models were implemented using the following trait-based Bayes-C0 model (Kizilkaya et al., 2010), noting that Bayes-C0 is equivalent to GBLUP (Strandén and Garrick, 2009):

$$y_{ij}={HYQ}_{ij}+\sum_{n=1}^{p}m_{ijn}\beta_{jn}+e_{ij}$$

where $y_{ij}$ is the binary POP phenotype (0/1) of sow *i* in parity *j* (*j* = 2–6); ${HYQ}_{ij}$ is the fixed contemporary group effect for sow *i* in parity *j,* as defined previously; $m_{ijn}$ is the genotype of sow *i* at SNP *n*, coded as 0, 1, and 2; $\beta_{jn}$ is the allele substitution effect of SNP *n* for parity *j*, where, for bivariate analysis of parities *j = k* and *j = l*, $\left[\begin{array}{c} \beta_{kn}\\ \beta_{ln} \end{array}\right]∼\;MVN\left(0,G\right)$ , where G is a 2 x 2 covariance matrix for the effects of SNPs for parities *k* and *l*, following Cheng et al. (2018); and $e_{ij}$ is the residual effect, with $\left[\begin{array}{c} e_{ik}\\ e_{il} \end{array}\right]∼\;MVN\left(0,R\right)$ , where R is a 2 x 2 covariance matrix of residual effects for a sow’s POP phenotypes for parities *k* and *l*.

The bivariate analyses were implemented in the Julia for Whole-genome Analysis Software (JWAS) (Cheng et al., 2018), using a Monte Carlo Markov chain of 120,000 iterations, with the initial 20,000 samples discarded as burn-in. For each 100th iteration, the sampled genome-wide genetic variances and covariances were saved and used to compute samples of the posterior distribution of the genetic correlation by dividing the sampled genome-wide genetic covariance by the product of the sampled genetic standard deviation for each trait. For inference, the mean and the 95% highest posterior density (HPD) interval of the posterior distribution of the genetic correlation were obtained using the ‘coda’ package in R (Plummer et al., 2015).

### 2.3 Genome-wide association study

To identify genomic regions associated with susceptibility to POP, the following univariate marker-based Bayes-B threshold model (Sorensen et al., 1995) was implemented for genome-wide association studies (GWAS) based on the across-parity and by-parity data, using JWAS (Cheng et al., 2018):

$$Probit(y_{ij})={HYQ}_{ij}+\sum_{n=1}^{p}m_{ijn}\beta_{jn}\delta_{jn}+e_{ij}$$

with effects as described above for the bivariate linear model for parity *j*, or the across-parity analysis, except for the addition of $\delta_{jn}$ , which indicates whether SNP *n* was (*δ*_*n*_ = 1) or was not (*δ*_*n*_ = 0) included in the model in that iteration of the chain, with the prior probability of inclusion (*π*) estimated using a Bayes-Cπ model (Kizilkaya et al., 2010). For the across-parity analyses, parity was included as another fixed effect. The residual variance was set to one, as it is not identifiable in threshold models. Inferences were based on a Monte Carlo Markov chain length of 50,000, with the first 5,000 iterations discarded as burn-in. Sample breeding values across the genome and for every 1 Mb non-overlapping window of the reference genome *Sus Scrofa* 11.1 from every 100th iteration were used to obtain samples of the posterior distributions of the genome-wide and window-based genetic variances and to estimate window-based posterior probabilities of association (WPPA), as described by Fernando et al. (2017).

Genomic regions associated with susceptibility to POP were identified using the across-parity analysis as 1 Mb windows that explained more than 1% of the genetic variance. The by-parity GWAS were used to confirm the across-parity GWAS results based on the presence of signals in the identified QTL regions in multiple parities. To allow for linkage disequilibrium extending over longer regions, which can result in the location of signals to differ between data sets, signals from the by-parity analyses were allowed to extend 2 Mb to either side of the QTL regions that were identified in the across-parity analyses. Thus, in the by-parity analyses, the signal in each by-parity analysis was quantified as the sum of the genetic variances estimated for the QTL region identified in the across-parity analysis plus the genetic variances estimated for the two 1 Mb windows upstream and downstream from that region, taken as a % of the genome-wide genetic variance.

### 2.4 Functional analyses

To obtain a better understanding of the QTL regions identified in the across-parity GWAS and their possible biological connections with susceptibility to POP, we identified the SNP that had the highest posterior probability of association (PPA, Fernando et al., 2017) in each region and used it as input for the pig Combined Annotation-Dependent Depletion (pCADD) pipeline developed by Derks et al. (2021). The pCADD pipeline utilizes the location of the input SNP to identify SNPs from whole genome sequence data that are in high linkage disequilibrium with the input SNP. These variants are then scored based on their impact (either regulatory or coding) and putative deleteriousness in relation to the trait. The pCADD scores are log-rank scores relative to the investigated SNP, ranging from 0 to 95 (Gross et al., 2020). A higher score indicates a greater likelihood of the variant having a functional impact. Scores higher than 20 and 30 are in the top 1% and 0.1%, respectively, of the highest scored SNPs (Gross et al., 2020; Derks et al., 2021). In addition, the Ensembl genome browser was used as a search and analysis tool to obtain information on genes located in the identified QTL regions (Cunningham et al., 2022).

Additional insight into biological pathways that contribute to susceptibility to POP was obtained by performing gene set enrichment analyses on results from the by-parity GWAS, following Cheng et al. (2022). For this purpose, results for non-overlapping 1 Mb windows from the five by-parity GWAS analyses were combined, ranked based on % of genetic variance explained, and annotated using the gene ontology (GO) database and using a pig transcriptome library. As described by Cheng et al. (2022), the GO data base library was adapted from the Molecular Signature Database (MSigDB) and consisted of curated gene sets that are annotated by the same ontology term (Subramanian et al., 2005). The pig transcriptomic library consisted of annotated gene sets that were derived from *in vivo* transcriptomic data for a variety of (patho) physiological states of different porcine tissue types (van Renne et al., 2018). Using both of these libraries in turn, a ranked gene set enrichment analysis was conducted using GSEA 4.2.3 (Subramanian et al., 2005).

## 3 Results

**FIGURE 1:**
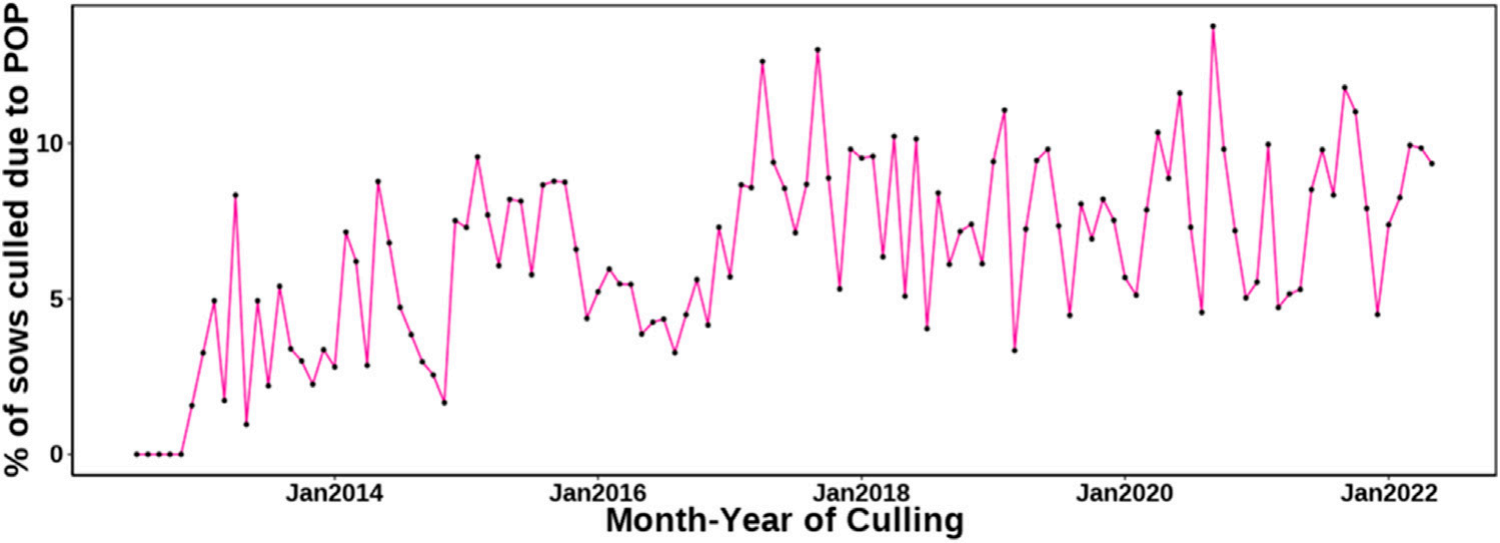
Percentage of sows culled due to pelvic organ prolapse (POP) as a function of culling month and year.

**FIGURE 2:**
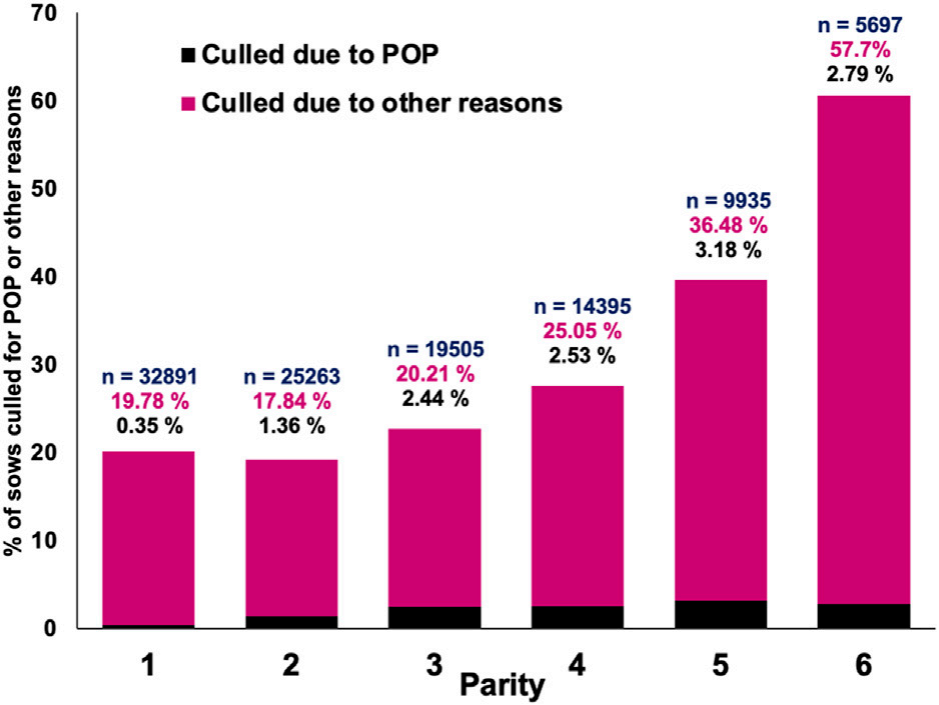
Percentage of sows that died or were culled due to pelvic organ prolapse (POP) or culled/died for other reasons by parity, where n denotes the total number of sow records present in that parity.

In total, 7.1% of the sows that were culled during the evaluated period were removed from the herd because of POP. The incidence of POP over time, as a percentage of sows that died or that were culled, is shown in Figure 1. It is evident that culling due to POP generally increased over time but with no obvious seasonal pattern. Figure 2 shows the percentage of sows present that were culled for POP *versus* other reasons by parity. The incidence of POP was low for parity 1 (0.35%) and then gradually increased to up to 3.18% of all sows present for parity 5. Parity 1 data were not used for analysis because of the low incidence. In the across-parity data used for analyses (parities 2-6), 10.1% of all culls were for POP.

### 3.1 Genetic parameters

For the across-parity analysis, the estimate of heritability of susceptibility to POP based on the logistic regression model was 0.21 ± 0.02 when using pedigree relationships and 0.35 ± 0.02 when using genomic relationships. These estimates are on the underlying scale and, based on the 10.1% incidence of culling due to POP, translate to estimates of 0.07 and 0.12, respectively, on the observed scale, following Falconer and Mackay (1996). Estimates of heritability by parity are in Table 1 and confirmed the moderate heritability of POP on the underlying scale that was obtained in the across-parity analysis. Estimates of heritability were higher and had lower SE when genomic instead of pedigree-based relationships were used.

**TABLE 1:** Estimates of genetic parameters of pelvic organ prolapse (POP). Estimates of heritability (±SE) based on logit models are on the diagonal (estimates using pedigree on first line; estimates using genomic relationships in bold on second line; estimates using Bayes-B analysis on third line). Off-diagonals are posterior means of genetic correlations between parities (below diagonal) and Lower/upper bounds for 95% Highest Posterior Density (HPD) intervals of genetic correlations (above diagonal) based on bivariate linear models using genomic relationships.

POP in:	Parity 2	Parity 3	Parity 4	Parity 5	Parity 6
Parity 2	0.27 ± 0.05	0.50—0.85	−0.01—0.83	0.15—0.83	−0.05—0.85
	**0.41 ± 0.03**				
	0.40 ± 0.02				
Parity 3	0.71	0.26 ± 0.04	0.37 – 0.85	0.14 – 0.82	−0.36 – 0.70
		**0.35 ± 0.03**			
		0.36 ± 0.02			
Parity 4	0.54	0.65	0.19 ± 0.05	0.48—0.87	−0.23—0.78
			**0.28 ± 0.04**		
			0.26 ± 0.02		
Parity 5	0.50	0.56	0.69	0.23 ± 0.05	−0.31—0.76
				**0.22 ± 0.04**	
				0.21 ± 0.02	
Parity 6	0.45	0.20	0.35	0.28	0.11 ± 0.09
					**0.15 ± 0.07**
					0.14 ± 0.02

The bold values are the estimates of heritability based on genomic relationships.

Estimates of genetic correlations of susceptibility to POP between parities were all positive (Table 1). Posterior distributions of the genetic correlations are in Supplementary Figure S1. Genetic correlation estimates were highest among neighboring parities and decreased with increasing distance between parities. The HPD intervals were also narrowest for some neighboring parity pairs (parities two to three and parities 4 and 5) and comparatively wide for correlations involving parity 6. Based on the 95% HPD intervals, most genetic correlations were significantly greater than 0, except genetic correlations of parity 2 with parity 4 and all correlations that involved parity 6. All genetic correlations were also significantly different from 1, with the upper bound of the HPD intervals ranging from 0.70 to 0.85.

### 3.2 Genomic regions associated with susceptibility to POP

**FIGURE 3:**
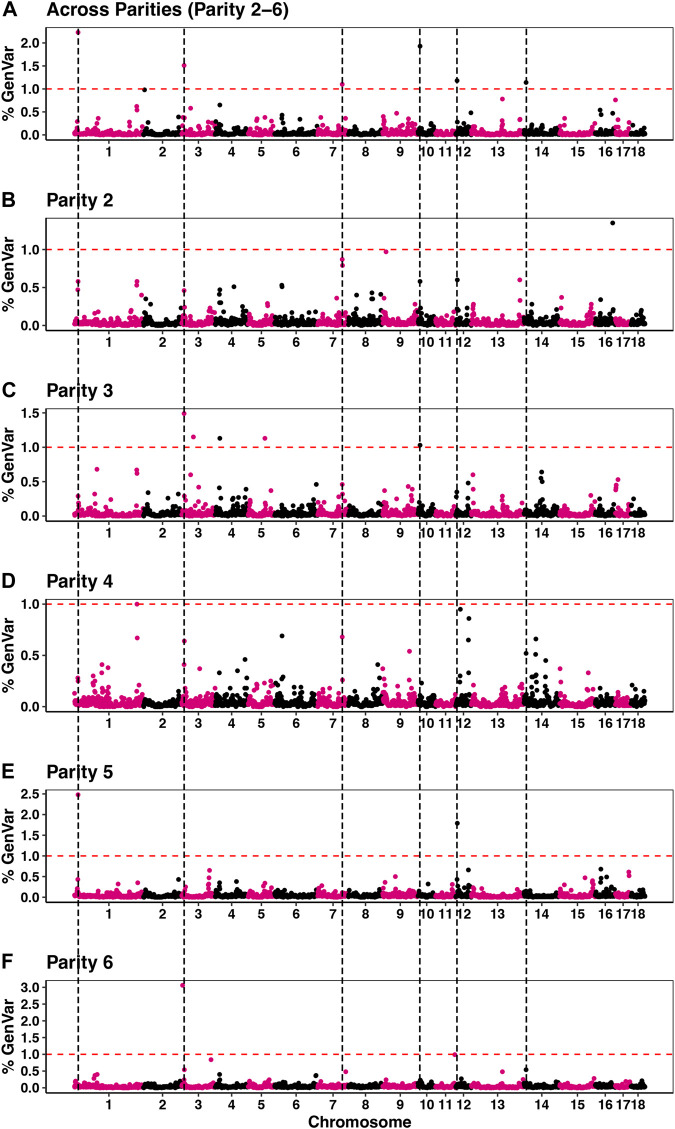
Percentage of genetic variance explained by non-overlapping 1 Mb windows across the genome for the across-parity **(A)** and by-parity **(B–F)** Bayes-B analyses. Windows (above the red dash line) were considered significant (explained >1% of genetic variance) and the black dashed lines identifies significant windows based on the across-parity analyses.

Estimates of *π* from the Bayes-Cπ analyses were 0.976 for the across-parity analysis and ranged from 0.983 to 0.992 for the by-parity analysis. These estimates were used in the corresponding Bayes-B analyses. Estimates of heritability based on the Bayes-B analyses were 0.36 **±** 0.01 for the across-parity analysis, while estimates for the by-parity analyses are reported in Table 1. Manhattan plots for GWAS of susceptibility to POP based on the across- and by-parity analyses are in Figure 3. A total of six windows that each explained more than 1% of the genetic variance were identified in the across-parity analysis and these are listed in Table 2. Combined, these 6 regions explained 9.0% of the genetic variance, with a region on SSC1 explaining 2.2%. Most of these regions had moderate to high WPPA, ranging from 0.79 to 0.99 for the region on SSC1, indicating substantial evidence that at least one SNP in these regions has a non-zero effect. Posterior inclusion probabilities (PIP) for each SNP in each region are reported in Supplementary Figure S2. For each of these six regions, the number of SNPs varied based on genotyping coverage on the SNP panel.

**TABLE 2:** Genomic regions that explained more than 1% of genetic variance per Mb for culling/death due to pelvic organ prolapse (POP) in the across-parity analyses and the corresponding % of genetic variance explained by that window ±2 Mb in the by-parity analyses, as well as potential candidate genes identified through annotated gene searches and pCADD analyses.

Chromo-some	Mb window	Number SNPs in window	Across-parity analysis		By parity analyses						Candidate genes
			WPPA*^a^*	% Of genetic variance	% Variance in window ±2 Mb						
					2	3		4	5	6	
**1**	14	43	0.99	2.23	1.15	0.65		0.63	3.23	0.31	*ESR1* ^*b*^ *, SYNE1* ^*c*^ *, CCDC170*
**3**	9	33	0.96	1.51	0.86	1.84		1.14	0.20	1.01	*COL26A1, RASA4B, ORAI2* ^*c*^ *, CUX1* ^*d*^
**7**	97	40	0.88	1.10	1.83	0.88		1.09	0.20	0.18	*LTBP2* ^*b*^
**10**	8	24	0.99	1.93	0.99	1.16		0.25	0.21	0.61	*TGFβ2* ^*b*^
**12**	5	22	0.90	1.18	0.96	0.6		0.27	2.41	0.31	*GGA3* ^*d*^ *, SMIM5* ^*c*^ *, ENSSSCG00000034198* ^*d*^
**14**	8	39	0.89	1.14	0.32	0.27		0.81	0.30	0.91	*STC1*

^*a*^WPPA, window posterior probability of association.

^*b*^ Genes identified through pCADD analysis with scores between 0 and 10.

^*c*^ Genes identified through pCADD analysis with scores between 10 and 20.

^*d*^ Genes identified through pCADD analysis with scores >20.

Table 2 presents the % of genetic variance that was explained in the by-parity analyses for the genomic regions that were identified in the across-parity analysis ±2 Mb on either side. Most genomic regions identified in the across-parity analyses explained more than 0.5% of the genetic variance in multiple by-parity analyses. The most significant 1 Mb window in the across-parity analyses did, however, not always coincide in location with the most significant 1 Mb window in the by-parity analyses, but it was generally close (Figure 3). Together, the genomic regions identified in the across-parity analyses explained 6.11, 5.4, 4, 19, 6.55, and 3.33% of the genetic variance in parities 2, 3, 4, 5, and 6, respectively, compared to 9.0% for the across-parity analysis.

### 3.3 Functional analyses

Through extensive candidate gene and pCADD analyses, several candidate genes were identified, as presented in Table 2. Genes *STC1* on SSC14 and *COL26A1* on SSC3 were identified as potential candidate genes based on genes annotated in the Ensemble genome browser. The pCADD analyses additionally unveiled several potential causal variants associated with susceptibility to POP. Genes *ESR1* on SSC1 and *TGFβ2* on SSC10 obtained a score between 0 and 10, while *SYNE1* on SSC1 and *ORAI2* on SSC3 obtained a pCADD score greater than 10. On SSC12, pCADD analysis revealed *GGA3, SMIM5,* and ENSSSCG00000034198 as genes with scores of 23, 20, and 23, respectively.

The GSEA analysis revealed several significant features that were enriched in the ranked 1 Mb windows, and these are listed in Table 3. For the GO term enrichment analysis, only GO terms enriched for susceptibility to POP at a q-value less than 0.2 are included in Table 3. For the porcine transcriptome enrichment library, only genes that were enriched at a q-value less than 0.05 are shown in Table 3 in order not to complicate results. Full results for both libraries are in the /Supplementary Materials. The QTL library developed by Cheng et al. (2022) was also used but did not result in any enriched QTL terms.

**TABLE 3:** Features that were significantly (q-value ≤0.05 for Porcine Transcriptome library and ≤0.2 for GO library) enriched in the ranked gene set enrichment analysis based on two customized libraries and the number of 1 Mb windows that contributed to the core.

Library	Enriched feature	q-value	# windows (total)^a^
Transcriptome	D5_Vs._D1_Wasting-Insensitive_Skeletal_Muscle_Acute_Quadriplegic_Myopathy_Model_Up	0.039	243 (750)
	D2_Vs._D0_Ileum_*Salmonella*_Typhimurium_Infection_Up	0.040	205 (795)
	P2_Vs._P0_Aortic_Valve_Interstitial_Cells_Up	0.041	185 (695)
	Iscom-Adjuvant_Vs._Ctrl_24h_Muscle_Tissue_Dn	0.043	189 (780)
	Ad_Lib_Vs._Fasted_Mc4r_D298_Adipose_Tissue_Dn	0.043	222 (730)
	Mature_Vs._Dedifferentiated_Adipocytes_Dn	0.044	231 (740)
	Electrosurgical_Incision_Vs._Non-Incised_Subcutaneous_Adipose_Tissue_Up	0.045	184 (705)
	Repetitive_Coronary_Stenosis_Vs._Ctrl_Myocardial_Infarction_Dn	0.048	190 (730)
	High_Prolificacy_Vs._Low_Prolificacy_Ovarian_Tissue_Up	0.049	155 (460)
	Infarct_Region_45d_Vs._Ctrl_Myocardial_Infarction_Up	0.050	203 (720)
	Infarct_Region_6d_Vs._Ctrl_Myocardial_Infarction_Up	0.050	176 (755)
Gene Ontology	Branch_Elongation_Involved_In_Mammary_Gland_Duct_Branching	0.088	5 (25)
	Antral_Ovarian_Follicle_Growth	0.129	8 (40)
	Estrogen_Receptor_Activity	0.200	4 (25)
	Prostate_Glandular_Acinus_Morphogenesis	0.194	6 (30)
	Mammary_Gland_Branching_Involved_In_Pregnancy	0.191	5 (25)
	Short_Chain_Fatty_Acid_Metabolic_Process	0.191	7 (20)
	Cation_Chloride_Symporter_Activity	0.199	(70) *
	Interleukin_1_Receptor_Binding	0.194	(35) *
	Determination_Of_Pancreatic_Left_Right_Asymmetry	0.194	(20) *
	Negative_Regulation_Of_Production_Of_Mirnas_Involved_In_Gene_Silencing_By_Mirna	0.189	(40) *
	Regulation_Of_Core_Promoter_Binding	0.184	(40) *
	Cellular_Response_To_Magnesium_Ion	0.189	(35) *
	Smooth_Muscle_Cell_Matrix_Adhesion	0.186	(25) *
	Regulation_Of_Synaptic_Transmission_Cholinergic	0.184	(30) *

^a^ # of windows contributing to the core of the enriched feature (total # windows with the feature) *Number in core not available.

## 4 Discussion

This study is the first to provide a detailed analysis of the genetic basis of pelvic organ prolapse in pigs. Using different types of analyses, this study clearly establishes a substantial role of genetics in susceptibility to POP and identifies several biological pathways that underlie the genetic basis of susceptibility to POP.

### 4.1 Genetic parameters

Heritability estimates are important statistics to measure the relative importance of genetic *versus* environmental influences on a trait and thus become pivotal in not only finding associated genomic regions but also developing selection strategies to reduce the prevalence of POP in sow herds. In contrast to our results, the only other previous study on the genetics of susceptibility to POP reported very low heritability estimates, indicating that environment and management are the major contributing factors for POP (Supakorn et al., 2019). Sows in that study, however, were from a different breed than used here and were kept in a different environment, potentially reflecting genetic differences or genotype by environment interactions. Initial analysis of the across-parity data of the present study using pedigree-based relationships indicated susceptibility to POP to be moderately heritable (Stevens et al., 2021; Dunkelberger et al., 2022). These estimates were confirmed here but using genomic relationships instead, as they provide more information on relationships among animals than pedigree and corrects and recovers missing pedigree information. Our estimates of heritability based on genomic relationships (Table 1) suggest that susceptibility to POP is, indeed, moderately heritable in the present data, 0.35 ± 0.02 based on the across-parity analysis, and thus can be selected against. The moderate estimate of heritability obtained from the across-parity analysis was validated by the by-parity analyses, which each provide partially independent information. The estimate of heritability was highest for parity 2 (0.41 ± 0.03) and decreased as parity number increased. Using genomic relationships resulted in significantly higher and more accurate estimates of heritability than using pedigree relationships, as quantified by their lower standard errors. The higher heritability estimates based on genomic versus pedigree-based relationships may be because of pedigree errors and missing sire information due to the use of pooled semen, both of which are expected to reduce pedigree-based estimates of heritability. All estimates in Table 1 are on the underlying logit scale and will be lower on the observed scale. The estimate of 0.35 on the logit scale based on the across-parity analysis translates to an estimate of 0.12 on the observed scale for the observed incidence of 10.1% in the across-parity data.

A trait can differ between parity of the sow with regards to its features or underlying causes. Parity differences may be the result of environmental effects but also of internal changes due to progression in age. From a genetics perspective, it is important to investigate whether the genetic basis of susceptibility to POP is similar across parities. Our moderate to high estimates of genetic correlations between parities (Table 1) suggest that susceptibility to POP indeed has a similar genetic basis across parities. Estimates of genetic correlations were moderate to strong positive for adjacent parities and decreased with distance between parities. Based on 95% HPD, all genetic correlations were significantly different from 0 and positive, except estimates that involved parity 6, likely because of the comparatively small sample size for this parity. All the genetic correlations were significantly different from 1 indicating that some differences in the genetic basis of susceptibility to POP may exist.

Further studies need to focus on understanding the genetic basis of susceptibility to POP in other genetic lines housed under different conditions, either in the United States or in other countries, to obtain a deeper understanding of the complexity of this trait and how it influences the overall incidence under the effect of different environment conditions. Additionally, our trait was defined as the occurrence of vaginal or uterine prolapse in sows; these traits were analysed together. In order to better understand the variation and biological processes involved with each prolapse condition (vaginal, uterine, or rectal), careful record collection is suggested to enable separate analysis of each diagnosis.

### 4.2 Genome-wide association analyses

Genomic analysis revealed several genomic regions that are associated with susceptibility to POP in the across-parity analysis, which included sows that were culled in parities 2 to 6 (Figure 3). We used the across-parity data to identify associated genomic regions, as it provides the most comprehensive data on susceptibility to POP. Depending on severity, prolapsed sows are either euthanized or are isolated for treatment. A comparison to sows that are culled for reasons other than POP provides, therefore, a logical approach to evaluate susceptibility to POP based on the available data. The across-parity analysis showed susceptibility to POP to be associated with regions on chromosomes 1, 3, 7, 10, 12, and 14. In addition to differences in WPPA between these genomic regions, extensive differences were also observed in the PIP for each marker within each region. Supplementary Figure S2 reports the patterns of PIP for SNPs in the identified regions. Except for the region on SSC3, all regions show only one or two sharp peaks in PIP, indicating strong associations of individual SNPs with the causative locus or loci. For the region on SSC3, a combination of SNPs jointly contributed to the overall effect of the region.

To further strengthen our understanding of these associations, we also performed GWAS using the by-parity data. Comparing the resulting Manhattan plots in Figure 3, most regions identified in the across-parity analysis were also identified in several by-parity analyses, although sometimes in a neighbouring 1 Mb region, likely because of the presence of extensive linkage disequilibrium and randomness. Because of this, the significant 1 Mb regions identified in the across-parity analysis were extended to 2 Mb on either side when quantifying the proportion of genetic variance explained in the by-parity analyses. Results in Table 2 showed that the genomic regions identified in the across-parity analysis explained at least 0.5% of genetic variance in multiple by-parity analyses. This partially independent validation not only reassured the importance of the significant regions found in the across-parity analysis, but also revealed other regions that may be of importance (e.g., the region on SSC16 for parity 2). The by-parity analyses also confirmed the similarity of the genetic basis of susceptibility to POP across parities, consistent with the moderately high estimates of genetic correlations between parities, but also hints at some differences in the susceptibility to POP between parities, possibly as a result of differences in the expression of genes.

The identified genomic region on SSC1 harbours several candidate genes, including the Estrogen Receptor 1 gene (*ESR1*), the Spectrin Repeat Containing Nuclear Envelope Protein 1 gene (*SYNE1*)*,* and the Coiled-coil Domain Containing 170 gene (*CCDC170*). In goats, *SYNE1* has been reported to be differentially expressed between animals with low and high litter yields and has been associated with biological processes such as ovarian follicle development and the ovulation cycle process (Liu et al., 2021). The *ESR1* gene was previously identified as a major gene affecting litter size in pigs (Rothschild et al., 1996; Goliášová and Wolf, 2004; Wu et al., 2006). As the name suggests, *ESR1* encodes an estrogen receptor that binds to estrogen, which also affects the expression of other genes (Charpentier et al., 2000). The *ESR1* gene primarily controls estrogen-mediated actions in female reproductive organs, and knockout mouse models have demonstrated its quintessential role in female reproductive tract development (Miyagawa and Iguchi, 2015). Chen et al. (2008) identified an association of an *ESR1* polymorphism with POP risk in a case control study in humans. In a recent study in pigs, increased abundance of estrogen precursors (androstenedione and androsterone) and other forms of estrogen (estrone and 17β-estradiol) were reported in sows with high *versus* low POP risk (Kiefer et al., 2021a). Moreover, several studies in humans have suggested changes in *ESR1* gene expression levels to be associated with POP risk (Zbucka-Kretowska et al., 2011; Wang et al., 2020), as it plays a supportive role in controlling the synthesis and breakdown of collagen in the pelvic region (Robinson and Cardozo, 2003).

Bone structure plays an important role in supporting pelvic organ integrity in mammals. These pelvic organs are held in place by a network of muscles, ligaments, and connective tissues that are anchored to pelvic bones, providing the essential support structure for the pelvic organs. Weakening or damage to this structure can result in misalignment of pelvic floor muscles, leading to protrusion of pelvic organs.

In humans, POP has been associated with low bone mass density (Ko et al., 2021) and post-menopausal women are at higher risk of osteoporosis due to estrogen deficiency (Ji and Yu, 2015). GWAS studies in humans have reported an association of the *CCDC170* gene with bone mineral density (Mullin et al., 2016), while other studies have confirmed the role of *CCDC170* in osteoporosis or hip fractures. (Hidalgo-Bravo et al., 2019). In laying hens, a SNP in the *CCDC170* gene was found to be associated with bone breaking strength (Jansen et al., 2021), solidifying the role of this gene in bone density across multiple species. Given the role of *ESR1* in regulating estrogen and the role of *CCDC170* in bone density and the close proximity of these two genes on SSC1, the conjunctive role of *CCDC170* and *ESR1* gene in POP risk in pigs needs to be further evaluated.

Several candidate genes that could be associated with susceptibility to POP are located in the identified associated genomic region on SSC3: Collagen Type XXVI Alpha 1 Chain (*COL26A1*), RAS P21 Protein Activator 4B (*RASA4B*)*,* and ORAI Calcium Release-Activated Calcium Modulator 2 (*ORAI2*). Collagen is the most abundant protein in vertebrates and is a major component of the extracellular matrix. Collagen is essential in providing strength and supportive functions to the pelvic floor (Gong and Xia, 2019) and collagen-related genes have been shown to be involved in pelvic floor support. *COL26A1* has also been found to play a role as an extracellular component in development of testis and ovaries (Sato et al., 2002). A differential gene expression study reported upregulation of *COL26A1* in pigs affected by scrotal hernia, indicating an altered collagen ratio that leads to weakened inguino-abdominal region support (Romano et al., 2020). The *RASA4B* gene has been reported to be important in the endometrium at 15 and 16 days of pregnancy in pigs (Kolakowska et al., 2017).

The *ORAI2* gene in the associated genomic region on SSC3 is a plasma membrane-related gene that plays a major role in maintaining calcium homeostasis, including in mature bovine corpora lutea (Wright et al., 2014; Trebak and Putney, 2017). Changes of calcium homeostasis are a major concern for smooth muscle functioning in the pelvic region (Adelstein and Sellers, 1987) and adequate provision of Ca is essential for bone formation, embryo implantation and development, and functioning of the placenta (Baczyk et al., 2011). After parturition in dairy cattle, hypocalcemia is highly prevalent (Reinhardt et al., 2011) and demand for calcium increases rapidly to support colostrum and milk synthesis (Horst et al., 2005). Pittman (2017) suggested hypocalcaemia to be one contributing factor to a sow’s higher susceptibility to POP. Requirements for calcium in sows increases greatly during late gestation (Mahan, 1990), as litter growth is physiologically demanding at that time (Kovacs, 2016) and, thus, improper diets can lead to inadequate levels of circulating calcium. Inadequate levels of circulating calcium can reduce bone strength and weaken overall bone structure, especially when pigs deplete their calcium reserves from bone to meet milk production demands (National Research Council, 1999; Crenshaw, 2000). In addition, deficient diets can also lead to higher incidence of skeletal problems, as the sow’s internal skeletal reserves are more affected (Maxson and Mahan, 1986).

The associated region on SSC3 also contains the Cut like Homeobox 1 gene (*CUX1*), which one study found to be a major candidate gene associated with calcium levels in blood in pigs (Reyer et al., 2019). In beef cattle, lower serum levels of calcium have been reported in uterine prolapsed animals (Richardson et al., 1981). Although various other studies have suggested hypocalcaemia to not be associated with risk of POP (Grez Capdeville, 2020; Grez-Capdeville and Crenshaw, 2020), they suggested the need to further understand phosphorus requirements, as its levels in blood are antagonistic to those of calcium and prolonged phosporus deprivation has been reported to cause muscle function disturbances in dairy cattle (Grünberg et al., 2019). Therefore, alterations in muscle function are another potential factor leading to higher risk of POP in sows.

The associated genomic region on SSC7 contains several QTL that have been reported for hind leg conformation (Le et al., 2017) and number of teats (Lopes et al., 2014) in pigs. This region also contains the Latent Transforming Growth Factor Beta-Binding Protein 2 (*LTBP2*) gene, which is a member of the Transforming Growth Factor β (TGF-β) latent complex and has been reported to bind Fibulin-5, which regulates elastic fibre assembly (Sideek et al., 2014). In humans, mutations in this gene have been found to cause syndromes that involve extracellular matrix disruptions (Haji-Seyed-Javadi et al., 2012). Interestingly, studies in knockout mice models have reported the crucial role of Fibulin-5 in pelvic organ support and disruptions in this protein can cause disordered homeostasis in elastic fibres, leading to POP (Drewes et al., 2007). Use of the SNP with the highest PIP in Supplementary Figure S2C for pCADD analysis also revealed the *LTBP2* gene as a potential candidate, with a score of 8.9 and 11.33 for two intronic variants for this gene.

The associated region on SSC10 contains the Transforming Growth Factor *β*2 (*TGFβ2*) gene, which belongs to the Transforming Growth Factor *β* superfamily and plays several important roles in uterine development and function based on studies in mice and human models (Li, 2014). Other studies in humans have also suggested the role of TGF*β* 1-3 isoforms in maternal support of embryo development (Jones et al., 2006) and regulation of expression and growth of uterine smooth muscle (Ciebiera et al., 2017).

The associated region on SSC12 harbors the Golgi Associated, Gamma Adaptin Ear Containing, ARF Binding Protein 3 (*GGA3*) gene, which was identified through the pCADD pipeline with a score of 23. This gene has been reported to play a role in cell migration and, more importantly, in regulating the levels of β1 integrins (Ratcliffe et al., 2016), which are key players in modulating extracellular matrix (ECM) and have been shown to be critical for contraction of collagen matrix (Schiro et al., 1991; Skinner et al., 1994; Lee et al., 1995). β1 integrins have also been reported to play a major role in upregulation of Matrix Metalloproteinase-9 (MMP-9) that degrades ECM and is shown to cause POP in fibulin-5 knockout mice models (Budatha et al., 2011). The pCADD pipeline also revealed the not annotated ENSSSCG00000034198 gene and the Small Integral Membrane Protein 5 (*SMIM5*) gene, with scores of 23 and 20, respectively. The *SMIM5* gene has been reported to encode structural proteins in skeletal muscle and has been found to be associated with marbling in cattle (Leal-Gutiérrez et al., 2019).

The associated region on SSC14 contains the Stanniocalcin-1 (*STC1*) gene, which codes for a glycoprotein that plays an important role in calcium/phosphorus homeostasis. Song et al. (2009) reported the *STC1* gene as an implantation marker in pigs, suggesting a strong biological role of this gene for uterine receptivity during implantation. In mice and sheep, studies have also reported a similar role of *STC1* gene in implantation (Stasko et al., 2001; Song et al., 2006). Moreover, the SSC14 region also harbours a QTL that has been reported for number of weaned piglets per litter (Suwannasing et al., 2018).

Our GWAS studies only utilized information from 48,075 genetic markers and future studies should focus on increasing the marker density for GWAS. This could result in more regions to be identified and in sharper PIP peaks, similar to those observed in Supplementary Figure S2.

### 4.3 Gene set enrichment analyses

The combined results of the by-parity analysed were used for enrichment analyses because it provided a larger number of partially independent observations on the relative importance of 1 Mb windows across the genome for the genetically correlated traits of susceptibility to POP by parity. The use of the by-parity analysis results for GSEA also provided partially independent confirmation of the genomic regions identified in the across-parity GWAS. While the GWAS for individual parities has limited power, their combined analysis in GSEA provides opportunities to identify biological processes that are associated with the trait of interest.

The GSEA analyses revealed several terms that were enriched for susceptibility to POP across the two investigated annotation libraries, as reported in Table 3. The GO library identified enrichment of genes associated with Estrogen Receptor Activity (q-value = 0.2), which confirmed our identification of the genomic region containing the *ESR1* gene in the across-parity GWAS. Furthermore, enrichment of the Antral Ovarian Follicle Growth GO term (q-value = 0.13) was observed, which overlaps with our finding from the candidate gene analysis that suggests a potential role of *SYNE1* gene in the identified region on SSC1. Genomic regions associated with POP were also enriched for the Interleukin (IL) Receptor 1 binding GO term (q-value = 0.19). Marcu et al. (2020) identified a role of IL1 in upregulation of matrix metalloproteinases that degrade extracellular matrix proteins. Interestingly, studies have also reported the role of these proteinases in regulation of mammary gland branching morphogenesis (Fata et al., 2004) and we observe enrichment of GO terms associated with Mammary Gland Branching during Pregnancy (q-value = 0.19) and Mammary Gland Duct Branching (q-value = 0.09).

## 5 Conclusion

This study provides multiple lines of evidence that susceptibility to POP is partially determined by genetics in the evaluated population and environment. We estimated moderate heritabilities in both the across- (35%) and by-parity analyses, moderately high estimates of genetic correlations of susceptibility to POP in the by-parity analysis, which confirms a similar genetic basis for POP across parities, and evidence of genomic regions associated with susceptibility to POP. The latter revealed several genomic regions to be associated with susceptibility to POP, in particular on chromosomes 1, 3, 7, 10, 12, and 14. Several candidate genes that could contribute to sow susceptibility to POP were identified, as well as various biological processes and pathways. These results suggest that, for this population and for the environment that prevailed for the evaluated sows, genetic selection can be used to reduce the incidence of POP. This, however, must be validated for other populations and environments, including investigation of possible genotype by environment interactions. In addition, knowledge of these identified biological pathways can be used to develop targeted management recommendations or interventions to reduce the risk of POP. Thus, results from this study provide important leads for potential solutions to a major industry-wide issue.

## Data Availability

The datasets analyzed for the current study include confidential information of the company that supplied these data. Therefore, these datasets are not publicly available. Relevant data can be made available upon reasonable request. Please direct requests to the corresponding author.
