# Supplementary material for: Identification of the genetic basis of sow pelvic organ prolapse
Source: Front Genet. 2023 Apr 18;14:1154713. doi: 10.3389/fgene.2023.1154713 (PMC10151575; doi:10.3389/fgene.2023.1154713)
Supplement: Supplementary file 1 [file DataSheet1.zip › Supplemental_Material/GSEA_Transcriptome.pdf]

Table: Gene sets enriched in phenotype **na** [plain text format]

	GS<br>follow link to MSigDB	GS DETAILS	SIZE	ES	NES
1	GSE24889_STREPTOCOCCUS_SUIS-INFECTED_VS_CTRL_24HPI_BRAIN_TISSUE_DN	Details ...	590	0.48	1.27
2	GSE22596_D2_VS_D0_ILEUM_SALMONELLA_TYPHIMURIUM_INFECTION_UP	Details ...	795	0.47	1.26
3	GSE13528_AD_LIB_VS_FASTED_MC4R_D298_ADIPOSE_TISSUE_DN	Details ...	730	0.47	1.25
4	GSE36306_ISCOM-ADJUVANT_VS_CTRL_24H_MUSCLE_TISSUE_DN	Details ...	780	0.47	1.24
5	GSE24239_D5_VS_D1_WASTING-INSENSITIVE_SKELETAL_MUSCLE_ACUTE_QUADRIPLEGIC_MYOPATHY_MODEL_UP	Details ...	750	0.47	1.24
6	GSE17264_MATURE_VS_DEDIFFERENTIATED_ADIPOCYTES_DN	Details ...	740	0.47	1.23
7	GSE48839_P2_VS_P0_AORTIC_VALVE_INTERSTITIAL_CELLS_UP	Details ...	695	0.47	1.23
8	GSE34569_INFARCT_REGION_45D_VS_CTRL_MYOCARDIAL_INFARCTION_UP	Details ...	755	0.46	1.22
9	GSE34569_INFARCT_REGION_6D_VS_CTRL_MYOCARDIAL_INFARCTION_UP	Details ...	720	0.46	1.22
10	GSE47710_ELECTROSURGICAL_INCISION_VS_NON-INCISED_SUBCUTANEOUS_ADIPOSE_TISSUE_UP	Details ...	705	0.46	1.22
11	GSE21096_REPETITIVE_CORONARY_STENOSIS_VS_CTRL_MYOCARDIAL_INFARCTION_DN	Details ...	730	0.46	1.22
12	GSE21383_HIGH_PROLIFICACY_VS_LOW_PROLIFICACY_OVARIAN_TISSUE_UP	Details ...	460	0.46	1.21
13	GSE22596_D2_VS_D0_ILEUM_SALMONELLA_TYPHIMURIUM_INFECTION_DN	Details ...	735	0.46	1.21
14	GSE12705_EARLY_FILAMENTOUS_VS_LATE_FILAMENTOUS_EMBRYONIC_TROPHOBLAST_ELONGATION_UP	Details ...	685	0.46	1.21
15	GSE73088_LETHAL_DOSE_MUSTARD_GAS_VS_CTRL_LUNG_TISSUE_UP	Details ...	725	0.46	1.20
16	GSE18467_LISSENCEPHALIC_VS_GYRENCEPHALIC_CEREBRAL_CORTEX_UP	Details ...	760	0.45	1.20
17	GSE21663_5HT_AND_FLUOXETIN_TREATED_VS_UNTREATED_CYCLICALLY-STRETCHED_AORTIC_VALVE_UP	Details ...	750	0.45	1.19
18	GSE15256_ANTIBIOTIC-TREATED_ISOLATOR-HOUSED_VS_INDOOR-HOUSED_WEANING-AGE_JEJUNUM_DN	Details ...	795	0.45	1.19
19	GSE23503_RESVERATROL-SUPPLEMENTED_VS_UNSUPPLEMENTED_HIGH-FAT_DIET_9MONTHS_PBMC_UP	Details ...	650	0.45	1.18
20	GSE23751_STREPTOCOCCUS_SUIS-INFECTED_VS_UNINFECTED_6HPI_CHOROID_PLEXUS_EPITHELIAL_CELLS_DN	Details ...	715	0.45	1.18
21	GSE26095_PROLIFERATIVE_VS_NON-PROLIFERATIVE_DEEP_WOUND_SKIN_CONES_20WK_UP		745	0.45	1.18
22	GSE22596_D1_VS_D0_COLON_SALMONELLA_TYPHIMURIUM_INFECTION_UP		740	0.44	1.18
23	GSE22596_D6_VS_D0_ILEUM_SALMONELLA_TYPHIMURIUM_INFECTION_DN		690	0.44	1.18

24	GSE13528_AD_LIB_VS_FASTED_MC4R_N298_LIVER_UP		760	0.44	1.18
25	GSE19083_PMWS_VS_HEALTHY_MEDIASTINAL_LYMPH_NODE_UP		715	0.45	1.18
26	GSE53997_JUVENILE_VS_ADULT_FAMILIAL_HYPERCHOLESTEROLEMIA_AORTIC_VALVE_INTERSTITIAL_CELLS_DN		660	0.45	1.18
27	GSE72025_SSEA1-NEG_VS_SSEA1-POS_FETAL_FIBROBLASTS_DN		620	0.45	1.18
28	GSE13528_AD_LIB_VS_FASTED_MC4R_D298_LIVER_UP		680	0.44	1.17
29	GSE23751_CAPSULE-DEFICIENT_STREPTOCOCCUS_SUIS-INFECTED_VS_UNINFECTED_6HPI_CHOROID_PLEXUS_EPITHELIAL_CELLS_DN		705	0.44	1.17
30	GSE21043_ATHEROSCLEROSIS_SUSCEPTIBLE_VS_ATHEROSCLEROSIS_PROTECTED_ARTERY_2YRS_UP		775	0.44	1.17
31	GSE14790_SUBCLINICAL_PCV2_VS_UNINFECTED_BLOOD_21DPI_UP		740	0.44	1.17
32	GSE48125_ANTIBIOTIC-TREATED_VS_CTRL_ILEUM_5D_POST_PREMATURE_BIRTH_UP		765	0.44	1.17
33	GSE30874_RESVERATROL-SUPPLEMENTED_VS_UNSUPPLEMENTED_ATHEROGENIC_DIET_4MONTHS_PBMC_DN		725	0.44	1.16
34	GSE22596_D6_VS_D0_COLON_SALMONELLA_TYPHIMURIUM_INFECTION_UP		640	0.44	1.16
35	GSE43072_MITRAL_INSUFFICIENCY_VS_NORMAL_ATRIAL_MYOCARDIAL_TISSUE_UP		450	0.44	1.15
36	GSE16348_D3_VS_D1_IMMOBILIZED_MUSCLE_DN		755	0.44	1.15
37	GSE13528_AD_LIB_VS_FASTED_MC4R_N298_ADIPOSE_TISSUE_DN		770	0.44	1.15
38	GSE26095_PROLIFERATIVE_VS_NON-PROLIFERATIVE_DEEP_WOUND_SKIN_CONES_20WK_DN		645	0.44	1.15
39	GSE19083_PMWS_VS_HEALTHY_MEDIASTINAL_LYMPH_NODE_DN		750	0.43	1.15
40	GSE30956_24H_VS_0H_LPS_STIMULATION_BONE-MORROW_DERIVED_MACROPHAGES_DN		715	0.43	1.15
41	GSE7314A_SALMONELLA_CHOLERAESUIS_INFECTED_VS_UNINFECTED_48HPI_MESENTERIAL_LYMPH_NODE_UP		710	0.43	1.15
42	GSE13528_MC4R_D298_VS_MC4R_N298_AD_LIB_LIVER_UP		610	0.43	1.15
43	GSE15256_ANTIBIOTIC-TREATED_ISOLATOR-HOUSED_VS_OUTDOOR-HOUSED_WEANING-AGE_JEJUNUM_DN		460	0.44	1.15
44	GSE15256_ANTIBIOTIC-TREATED_ISOLATOR-HOUSED_VS_OUTDOOR-HOUSED_NEAR-MATURE_JEJUNUM_DN		715	0.43	1.14
45	GSE27000_HIGH-SHEDDING_VS_LOW-SHEDDING_SALMONELLA_TYPHIMURIUM-INFECTED_2DPI_BLOOD_UP		755	0.43	1.14
46	GSE41636_LEAN_VS_OBESE_THORACIC_AORTA_DN		730	0.43	1.14
47	GSE23596_STREPTOCOCCUS_SUIS-INFECTED_VS_CTRL_SPLEEN_3DPI_DN		805	0.43	1.13

48	GSE21043_ATHEROSCLEROSIS_SUSCEPTIBLE_VS_ATHEROSCLEROSIS_PROTECTED_ARTERY_13D_UP		785	0.43	1.13
49	GSE53997_JUVENILE_VS_ADULT_FAMILIAL_HYPERCHOLESTEROLEMIA_AORTIC_VALVE_INTERSTITIAL_CELLS_UP		750	0.43	1.13
50	GSE22165_30MIN_METHYLENE_BLUE-TREATED_VS_CTRL_20MIN_POST_CARDIAC_ARREST_CEREBRAL_CORTEX_DN		725	0.43	1.13
51	GSE13528_MC4R_D298_VS_MC4R_N298_FASTED_ADIPOSE_TISSUE_UP		645	0.43	1.12
52	GSE21096_ISCHEMIC_PRECONDITIONED_VS_REPETITIVE_CORONARY_STENOSIS_MYOCARDIAL_INFARCTION_UP		755	0.42	1.12
53	GSE15472_INDUCED_PLURIPOTENT_STEM_CELLS_VS_FETAL_FIBROBLAST_CELLS_DN		730	0.42	1.12
54	GSE21071_CYSTIC_FIBROSIS_VS_HEALTHY_NEONATAL_BRONCHUS_DN		770	0.42	1.12
55	GSE13528_AD_LIB_VS_FASTED_MC4R_D298_ADIPOSE_TISSUE_UP		730	0.42	1.12
56	GSE49290_LOW_STRESS_VS_HIGH_STRESS_LIVER_TISSUE_DN		730	0.42	1.12
57	GSE7314A_SALMONELLA_CHOLERAESUIS_INFECTED_VS_UNINFECTED_48HPI_MESENTERIAL_LYMPH_NODE_DN		740	0.42	1.12
58	GSE21663_CYCLICALLY-STRETCHED_VS_STATIC_AORTIC_VALVE_UP		775	0.42	1.12
59	GSE64246_INACTIVATED_S_AUREUS-TREATED_3H_VS_UNTREATED_MAMMARY_EPITHELIAL_CELLS_UP		790	0.42	1.12
60	GSE12705_TUBULAR_VS_EARLY_FILAMENTOUS_EMBRYONIC_TROPHOBLAST_ELONGATION_UP		835	0.42	1.11
61	GSE12705_SPHERICAL_VS_TUBULAR_EMBRYONIC_TROPHOBLAST_ELONGATION_UP		675	0.42	1.11
62	GSE21043_ATHEROSCLEROSIS_SUSCEPTIBLE_VS_ATHEROSCLEROSIS_PROTECTED_ARTERY_2YRS_DN		865	0.42	1.11
63	GSE34569_INFARCT_REGION_30D_VS_CTRL_MYOCARDIAL_INFARCTION_UP		685	0.42	1.11
64	GSE13528_AD_LIB_VS_FASTED_MC4R_N298_ADIPOSE_TISSUE_UP		655	0.42	1.11
65	GSE22596_D1_VS_D0_JEJUNUM_SALMONELLA_TYPHIMURIUM_INFECTION_DN		690	0.42	1.11
66	GSE16348_D5_VS_D1_IMMOBILIZED_MUSCLE_UP		635	0.42	1.11
67	GSE47814_MODIFIED_GLENN_SHUNT-TREATED_VS_UNTREATED_RIGHT_VENTRICULAR_FAILURE_UP		765	0.42	1.11
68	GSE18359_AD_LIB_VS_CALORIE-RESTRICTED_HIGH_FEED_EFFICACY_LIVER_UP		815	0.42	1.11
69	GSE31191_LOW_VS_ADEQUATE_PROTEIN_DIET_FETAL_LIVER_TISSUE_DN		610	0.42	1.10
70	GSE15256_ANTIBIOTIC-TREATED_ISOLATOR-HOUSED_VS_OUTDOOR-HOUSED_NEAR-MATURE_JEJUNUM_UP		680	0.42	1.10
71	GSE7313A_SALMONELLA_TYPHIMURIUM_INFECTED_VS_UNINFECTED_24HPI_MESENTERIAL_LYMPH_NODE_DN		760	0.41	1.10

72	GSE13528_AD_LIB_VS_FASTED_MC4R_D298_LIVER_DN		770	0.41	1.10
73	GSE64246_INACTIVATED_E_COLI-TREATED_3H_VS_UNTREATED_MAMMARY_EPITHELIAL_CELLS_DN		730	0.42	1.10
74	GSE49290_LOW_STRESS_VS_HIGH_STRESS_LIVER_TISSUE_UP		765	0.41	1.10
75	GSE64246_INACTIVATED_E_COLI-TREATED_24H_VS_UNTREATED_MAMMARY_EPITHELIAL_CELLS_DN		710	0.41	1.10
76	GSE14790_SUBCLINICAL_PCV2_VS_UNINFECTED_BLOOD_7DPI_DN		800	0.41	1.10
77	GSE32956_63DPC_VS_91DPC_FETAL_MAMMARY_GLAND_DN		690	0.41	1.10
78	GSE19275_HIGH_FATTENING_VS_LOW_FATTENING_MUSCLE_TISSUE_DN		675	0.41	1.10
79	GSE18359_AD_LIB_VS_CALORIE-RESTRICTED_HIGH_FEED_EFFICACY_ADIPOSE_TISSUE_DN		725	0.41	1.10
80	GSE34569_NON-INFARCTED_REGION_30D_VS_CTRL_MYOCARDIAL_INFARCTION_DN		765	0.41	1.09
81	GSE33246_LOW_DOSE_VS_UNTREATED_DON-TREATED_BASOLATERAL_IPEC-J2_DN		705	0.41	1.09
82	GSE14758_SUBCLINICAL_PCV2_VS_UNINFECTED_MEDIASTINAL_LYMPH_NODE_29DPI_UP		730	0.41	1.09
83	GSE12705_TUBULAR_VS_EARLY_FILAMENTOUS_EMBRYONIC_TROPHOBLAST_ELONGATION_DN		720	0.41	1.09
84	GSE15211_NORMOTENSIVE_VS_HYPERTENSIVE_AORTIC_VALVE_INTERSTITIAL_CELLS_DN		760	0.41	1.09
85	GSE33246_HIGH_DOSE_VS_UNTREATED_DON-TREATED_BASOLATERAL_IPEC-J2_DN		715	0.41	1.08
86	GSE48125_ANTIBIOTIC-TREATED_VS_CTRL_DUODENUM_5D_POST_PREMATURE_C-SECTION_DN		760	0.41	1.08
87	GSE7313A_SALMONELLA_TYPHIMURIUM_INFECTED_VS_UNINFECTED_48HPI_MESENTERIAL_LYMPH_NODE_UP		705	0.41	1.08
88	GSE22782_D5_VS_D0_ALVEOLAR_MACROPHAGES_PRRSV_INFECTION_UP		720	0.41	1.08
89	GSE22165_30MIN_METHYLENE_BLUE-TREATED_VS_CTRL_20MIN_POST_CARDIAC_ARREST_CEREBRAL_CORTEX_UP		675	0.41	1.08
90	GSE24239_D5_VS_D1_WASTING-SENSITIVE_SKELETAL_MUSCLE_ACUTE_QUADRIPLEGIC_MYOPATHY_MODEL_UP		700	0.41	1.08
91	GSE22596_D2_VS_D0_COLON_SALMONELLA_TYPHIMURIUM_INFECTION_UP		690	0.41	1.08
92	GSE36306_ISCOM-ADJUVANT_VS_CTRL_24H_LYMPH_NODE_DN		835	0.41	1.08
93	GSE43072_MITRAL_INSUFFICIENCY_VS_NORMAL_ATRIAL_MYOCARDIAL_TISSUE_DN		430	0.41	1.08
94	GSE31191_LOW_VS_ADEQUATE_PROTEIN_DIET_YOUNG_LIVER_TISSUE_DN		760	0.41	1.08
95	GSE24239_D5_VS_D1_WASTING-INSENSITIVE_SKELETAL_MUSCLE_ACUTE_QUADRIPLEGIC_MYOPATHY_MODEL_DN		725	0.41	1.08

96	GSE21071_CYSTIC_FIBROSIS_VS_HEALTHY_NEONATAL_LUNG_DN		740	0.41	1.07
97	GSE7313A_SALMONELLA_TYPHIMURIUM_INFECTED_VS_UNINFECTED_21DPI_MESENTERIAL_LYMPH_NODE_UP		760	0.41	1.07
98	GSE32956_63DPC_VS_91DPC_FETAL_MAMMARY_GLAND_UP		800	0.41	1.07
99	GSE28003_STA-KO_STB-LOW_LT-KO_VS_WT_E_COLI_JEJUNUM_4HPI_UP		745	0.41	1.07
100	GSE33246_HIGH_DOSE_VS_UNTREATED_DON-TREATED_APICAL_IPEC-J2_DN		710	0.40	1.07
101	GSE23596_STREPTOCOCCUS_SUIS-INFECTED_VS_CTRL_SPLEEN_3DPI_UP		750	0.40	1.07
102	GSE14643_STEM-CELL_TREATED_VS_CTRL_MYOCARDIAL_INFARCTION_UP		845	0.40	1.07
103	GSE13528_MC4R_D298_VS_MC4R_N298_FASTED_ADIPOSE_TISSUE_DN		705	0.41	1.07
104	GSE12705_SPHERICAL_VS_TUBULAR_EMBRYONIC_TROPHOBLAST_ELONGATION_DN		790	0.40	1.07
105	GSE34569_INFARCT_REGION_6D_VS_CTRL_MYOCARDIAL_INFARCTION_DN		740	0.40	1.07
106	GSE13528_AD_LIB_VS_FASTED_MC4R_N298_LIVER_DN		675	0.40	1.07
107	GSE48125_ANTIBIOTIC-TREATED_VS_CTRL_DUODENUM_5D_POST_PREMATURE_C-SECTION_UP		735	0.40	1.06
108	GSE13528_MC4R_D298_VS_MC4R_N298_AD_LIB_LIVER_DN		480	0.40	1.06
109	GSE30956_7H_VS_0H_LPS_STIMULATION_BONE-MORROW_DERIVED_MACROPHAGES_UP		790	0.40	1.06
110	GSE21096_REPETITIVE_CORONARY_STENOSIS_VS_CTRL_MYOCARDIAL_INFARCTION_UP		750	0.40	1.06
111	GSE7313A_SALMONELLA_TYPHIMURIUM_INFECTED_VS_UNINFECTED_21DPI_MESENTERIAL_LYMPH_NODE_DN		660	0.40	1.06
112	GSE18854_D1_OF_DEDIFFERENTIATION_VS_D0_FOLLICULAR_GRANULOSA_CELLS_DN		790	0.40	1.06
113	GSE23751_CAPSULE-DEFICIENT_STREPTOCOCCUS_SUIS-INFECTED_VS_UNINFECTED_6HPI_CHOROID_PLEXUS_EPITHELIAL_CELLS_UP		855	0.40	1.06
114	GSE23503_RESVERATROL-SUPPLEMENTED_VS_UNSUPPLEMENTED_HIGH-FAT_DIET_9MONTHS_PBMC_DN		740	0.40	1.06
115	GSE21096_ISCHEMIC_PRECONDITIONED_VS_CTRL_MYOCARDIAL_INFARCTION_DN		705	0.40	1.06
116	GSE48839_TGF1BETA-TREATED_VS_UNTREATED_24H_AORTIC_VALVE_INTERSTITIAL_CELLS_UP		785	0.40	1.05
117	GSE18359_AD_LIB_VS_CALORIE-RESTRICTED_LOW_FEED_EFFICACY_ADIPOSE_TISSUE_DN		705	0.40	1.05
118	GSE18854_D2_OF_DEDIFFERENTIATION_VS_D0_FOLLICULAR_GRANULOSA_CELLS_UP		740	0.40	1.05
119	GSE14643_STEM-CELL_TREATED_VS_CTRL_MYOCARDIAL_INFARCTION_DN		565	0.40	1.05

120	GSE17492_BRUCELLA_SUIS-INFECTED_VS_UNINFECTED_SPLEEN_DN		695	0.40	1.05
121	GSE28003_WT_VS_CTRL_E_COLI_JEJUNUM_4HPI_DN		825	0.40	1.05
122	GSE44326_HIGH_VS_LOW_PROTEIN_DIET_MUSCLE_TISSUE_DN		480	0.40	1.05
123	GSE47814_MODIFIED_GLENN_SHUNT-TREATED_VS_UNTREATED_RIGHT_VENTRICULAR_FAILURE_DN		735	0.40	1.05
124	GSE22596_D1_VS_D0_COLON_SALMONELLA_TYPHIMURIUM_INFECTION_DN		605	0.40	1.05
125	GSE17492_BRUCELLA_SUIS-INFECTED_VS_UNINFECTED_SPLEEN_UP		705	0.40	1.05
126	GSE12705_EARLY_FILAMENTOUS_VS_LATE_FILAMENTOUS_EMBRYONIC_TROPHOBLAST_ELONGATION_DN		760	0.39	1.04
127	GSE44326_HIGH_VS_LOW_PROTEIN_DIET_MUSCLE_TISSUE_UP		740	0.39	1.04
128	GSE7314A_SALMONELLA_CHOLERAESUIS_INFECTED_VS_UNINFECTED_21DPI_MESENTERIAL_LYMPH_NODE_UP		860	0.39	1.04
129	GSE65008_SUCKLING_VS_WEANED_35DPN_ILEAL_MUCOSA_UP		715	0.39	1.04
130	GSE47814_RIGHT_VENTRICULAR_FAILURE_VS_NORMAL_MYOCARDIAL_TISSUE_DN		715	0.39	1.04
131	GSE36306_ISCOM-ADJUVANT_VS_CTRL_24H_LYMPH_NODE_UP		765	0.39	1.04
132	GSE31191_LOW_VS_ADEQUATE_PROTEIN_DIET_FETAL_LIVER_TISSUE_UP		770	0.39	1.04
133	GSE26663_SEDENTARY_VS_EXERCISE-TRAINED_BRACHIAL_ARTERY_ENDOTHELIUM_DN		750	0.39	1.04
134	GSE37166_CORTICOSTEROID-<br>TREATED_VS_UNTREATED_SKELETAL_MUSCLE_ACUTE_QUADRIPLEGIC_MYOPATHY_MODEL_DN		620	0.39	1.04
135	GSE8974A_YOUNG_VS_SENESCENT_ENDOTHELIAL_CELLS_UP		655	0.39	1.04
136	GSE64246_INACTIVATED_S_AUREUS-TREATED_24H_VS_UNTREATED_MAMMARY_EPITHELIAL_CELLS_DN		700	0.39	1.03
137	GSE15472_INDUCED_PLURIPOTENT_STEM_CELLS_VS_FETAL_FIBROBLAST_CELLS_UP		715	0.39	1.03
138	GSE21383_HIGH_PROLIFICACY_VS_LOW_PROLIFICACY_OVARIAN_TISSUE_DN		525	0.39	1.03
139	GSE19275_HIGH_FATTENING_VS_LOW_FATTENING_MUSCLE_TISSUE_UP		775	0.39	1.03
140	GSE22782_D5_VS_D0_ALVEOLAR_MACROPHAGES_PRRSV_INFECTION_DN		740	0.39	1.03
141	GSE47710_ELECTROSURGICAL_INCISION_VS_NON-INCISED_SUBCUTANEOUS_ADIPOSE_TISSUE_DN		785	0.39	1.03
142	GSE21071_CYSTIC_FIBROSIS_VS_HEALTHY_NEONATAL_LUNG_UP		810	0.39	1.03
143	GSE21043_ATHEROSCLEROSIS_SUSCEPTIBLE_VS_ATHEROSCLEROSIS_PROTECTED_ARTERY_9MONTHS_UP		815	0.39	1.03

144	GSE36306_ISCOM-ADJUVANT_VS_CTRL_24H_MUSCLE_TISSUE_UP		740	0.39	1.03
145	GSE26663_SEDENTARY_VS_EXERCISE-TRAINED_BRACHIAL_ARTERY_ENDOTHELIUM_UP		720	0.39	1.03
146	GSE22596_D2_VS_D0_COLON_SALMONELLA_TYPHIMURIUM_INFECTION_DN		765	0.39	1.03
147	GSE21663_CYCLICALLY-STRETCHED_VS_STATIC_5HT_AND_FLUOXETIN_TREATED_AORTIC_VALVE_UP		760	0.39	1.02
148	GSE22487_D7_VS_D0_NEONATAL_SKELETAL_MUSCLE_UP		760	0.39	1.02
149	GSE15256_ANTIBIOTIC-TREATED_ISOLATOR-HOUSED_VS_INDOOR-HOUSED_NEONATAL_JEJUNUM_UP		680	0.39	1.02
150	GSE17264_MATURE_VS_DEDIFFERENTIATED_ADIPOCYTES_UP		840	0.38	1.02
151	GSE24762_REGENERATED-DERIVED_VS_NATIVE-DERIVED_ENDOTHELIAL_CELLS_DN		450	0.39	1.02
152	GSE24762_REGENERATED-DERIVED_VS_NATIVE-DERIVED_ENDOTHELIAL_CELLS_UP		785	0.38	1.02
153	GSE21096_ISCHEMIC_PRECONDITIONED_VS_REPETITIVE_CORONARY_STENOSIS_MYOCARDIAL_INFARCTION_DN		795	0.39	1.02
154	GSE37922_EXERCISE_VS_SEDENTARY_MYOCARDIAL_TISSUE_UP		690	0.38	1.02
155	GSE16348_D3_VS_D1_IMMOBILIZED_MUSCLE_UP		715	0.38	1.02
156	GSE21096_ISCHEMIC_PRECONDITIONED_VS_CTRL_MYOCARDIAL_INFARCTION_UP		705	0.38	1.02
157	GSE21096_REPETITIVE_CORONARY_OCCLUSION_VS_CTRL_MYOCARDIAL_INFARCTION_UP		650	0.39	1.02
158	GSE18359_AD_LIB_VS_CALORIE-RESTRICTED_LOW_FEED_EFFICACY_LIVER_UP		775	0.39	1.02
159	GSE66317_LOW_LACTOFERRIN-SUPPLEMENTED_VS_STANDARD_DIET_JUVENILE_HIPPOCAMPUS_UP		780	0.39	1.02
160	GSE7313A_SALMONELLA_TYPHIMURIUM_INFECTED_VS_UNINFECTED_48HPI_MESENTERIAL_LYMPH_NODE_DN		755	0.38	1.02
161	GSE7313A_SALMONELLA_TYPHIMURIUM_INFECTED_VS_UNINFECTED_24HPI_MESENTERIAL_LYMPH_NODE_UP		755	0.38	1.01
162	GSE18343_PSEUDOPREGNANT_VS_ENDOCRINE_DISRUPTED_ENDOMETRIUM_D13_DN		710	0.38	1.01
163	GSE30956_24H_VS_0H_LPS_STIMULATION_BONE-MORROW_DERIVED_MACROPHAGES_UP		715	0.38	1.01
164	GSE41636_LEAN_VS_OBESE_THORACIC_AORTA_UP		820	0.38	1.01
165	GSE21663_5HT_AND_FLUOXETIN_TREATED_VS_UNTREATED_CYCLICALLY-STRETCHED_AORTIC_VALVE_DN		695	0.38	1.01
166	GSE26663_SEDENTARY_VS_EXERCISE-TRAINED_INTERNAL_MAMMARY_ARTERY_ENDOTHELIUM_UP		750	0.38	1.01
167	GSE21043_ATHEROSCLEROSIS_SUSCEPTIBLE_VS_ATHEROSCLEROSIS_PROTECTED_ARTERY_9MONTHS_DN		850	0.38	1.01

168	GSE18854_D2_OF_DEDIFFERENTIATION_VS_D0_FOLLICULAR_GRANULOSA_CELLS_DN		825	0.38	1.01
169	GSE18854_D4_OF_DEDIFFERENTIATION_VS_D0_FOLLICULAR_GRANULOSA_CELLS_DN		790	0.38	1.01
170	GSE15256_ANTIBIOTIC-TREATED_ISOLATOR-HOUSED_VS_INDOOR-HOUSED_WEANING-AGE_JEJUNUM_UP		775	0.38	1.01
171	GSE21663_CYCLICALLY-STRETCHED_VS_STATIC_AORTIC_VALVE_DN		790	0.38	1.01
172	GSE22487_D14_VS_D0_NEONATAL_SKELETAL_MUSCLE_UP		770	0.38	1.01
173	GSE18359_AD_LIB_VS_CALORIE-RESTRICTED_HIGH_FEED_EFFICACY_ADIPOSE_TISSUE_UP		785	0.38	1.01
174	GSE23751_STREPTOCOCCUS_SUIS-INFECTED_VS_UNINFECTED_6HPI_CHOROID_PLEXUS_EPITHELIAL_CELLS_UP		790	0.38	1.01
175	GSE18359_HIGH_VS_LOW_FEED_EFFICACY_AD_LIB_ADIPOSE_TISSUE_DN		615	0.38	1.01
176	GSE18359_AD_LIB_VS_CALORIE-RESTRICTED_HIGH_FEED_EFFICACY_LIVER_DN		725	0.38	1.01
177	GSE48125_ANTIBIOTIC-TREATED_VS_CTRL_ILEUM_5D_POST_PREMATURE_BIRTH_DN		725	0.38	1.00
178	GSE24889_STREPTOCOCCUS_SUIS-INFECTED_VS_CTRL_24HPI_PBMC_UP		615	0.38	1.00
179	GSE21663_CYCLICALLY-STRETCHED_VS_STATIC_5HT_AND_FLUOXETIN_TREATED_AORTIC_VALVE_DN		680	0.38	1.00
180	GSE22487_D21_VS_D0_NEONATAL_SKELETAL_MUSCLE_UP		715	0.38	1.00
181	GSE18343_PSEUDOPREGNANT_VS_ENDOCRINE_DISRUPTED_ENDOMETRIUM_D13_UP		465	0.38	1.00
182	GSE22596_D1_VS_D0_ILEUM_SALMONELLA_TYPHIMURIUM_INFECTION_UP		715	0.38	1.00
183	GSE18359_AD_LIB_VS_CALORIE-RESTRICTED_LOW_FEED_EFFICACY_ADIPOSE_TISSUE_UP		725	0.38	1.00
184	GSE34569_NON-INFARCTED_REGION_30D_VS_CTRL_MYOCARDIAL_INFARCTION_UP		745	0.38	0.99
185	GSE14758_SUBCLINICAL_PCV2_VS_UNINFECTED_MEDIASTINAL_LYMPH_NODE_29DPI_DN		770	0.38	0.99
186	GSE22596_D1_VS_D0_JEJUNUM_SALMONELLA_TYPHIMURIUM_INFECTION_UP		670	0.38	0.99
187	GSE33037_ENDOTOXIN-TREATED_VS_UNTREATED_SKELETAL_MUSCLE_ACUTE_QUADRIPLEGIC_MYOPATHY_MODEL_UP		765	0.38	0.99
188	GSE21043_ATHEROSCLEROSIS_SUSCEPTIBLE_VS_ATHEROSCLEROSIS_PROTECTED_ARTERY_13D_DN		875	0.37	0.99
189	GSE18854_D4_OF_DEDIFFERENTIATION_VS_D0_FOLLICULAR_GRANULOSA_CELLS_UP		780	0.37	0.99
190	GSE41636_LEAN_VS_OBESE_CORONARY_ARTERY_DN		725	0.37	0.99
191	GSE27000_HIGH-SHEDDING_VS_LOW-SHEDDING_SALMONELLA_TYPHIMURIUM-INFECTED_2DPI_BLOOD_DN		715	0.37	0.98

192	GSE22487_D14_VS_D0_NEONATAL_SKELETAL_MUSCLE_DN		665	0.37	0.98
193	GSE7314A_SALMONELLA_CHOLERAESUIS_INFECTED_VS_UNINFECTED_21DPI_MESENTERIAL_LYMPH_NODE_DN		795	0.37	0.98
194	GSE19975_WHITE_VS_RED_SKELETAL_MUSCLE_DN		840	0.37	0.98
195	GSE28003_STA-KO_STB-KO_LT-KO_VS_WT_E_COLI_JEJUNUM_4HPI_UP		775	0.37	0.98
196	GSE37922_EXERCISE_VS_SEDENTARY_MYOCARDIAL_TISSUE_DN		435	0.37	0.97
197	GSE21096_REPETITIVE_CORONARY_OCCLUSION_VS_CTRL_MYOCARDIAL_INFARCTION_DN		780	0.37	0.97
198	GSE8974A_YOUNG_VS_SENESCENT_ENDOTHELIAL_CELLS_DN		785	0.36	0.97
199	GSE22596_D2_VS_D0_JEJUNUM_SALMONELLA_TYPHIMURIUM_INFECTION_DN		765	0.37	0.97
200	GSE34569_INFARCT_REGION_45D_VS_CTRL_MYOCARDIAL_INFARCTION_DN		725	0.37	0.97
201	GSE21096_ISCHEMIC_PRECONDITIONED_VS_REPETITIVE_CORONARY_OCCLUSION_MYOCARDIAL_INFARCTION_UP		790	0.36	0.96
202	GSE18641_PREGNANT_VS_NON-PREGNANT_ENDOMETRIUM_D14_DN		665	0.36	0.96
203	GSE18641_PREGNANT_VS_NON-PREGNANT_ENDOMETRIUM_D14_UP		875	0.36	0.96
204	GSE64246_INACTIVATED_S_AUREUS-TREATED_3H_VS_UNTREATED_MAMMARY_EPITHELIAL_CELLS_DN		715	0.36	0.96
205	GSE64246_INACTIVATED_E_COLI-TREATED_3H_VS_UNTREATED_MAMMARY_EPITHELIAL_CELLS_UP		845	0.36	0.96
206	GSE47814_RIGHT_VENTRICULAR_FAILURE_VS_NORMAL_MYOCARDIAL_TISSUE_UP		750	0.36	0.96
207	GSE33246_LOW_DOSE_VS_UNTREATED_DON-TREATED_BASOLATERAL_IPEC-J2_UP		735	0.36	0.96
208	GSE30956_2H_VS_0H_LPS_STIMULATION_BONE-MORROW_DERIVED_MACROPHAGES_DN		680	0.36	0.96
209	GSE28003_STA-KO_STB-KO_LT-KO_VS_WT_E_COLI_JEJUNUM_4HPI_DN		725	0.36	0.95
210	GSE65008_SUCKLING_VS_WEANED_28DPN_ILEAL_MUCOSA_UP		770	0.36	0.95
211	GSE24239_WASTING-SENSITIVE_VS_WASTING-INSENSITIVE_SKELETAL_MUSCLE_ACUTE_QUADRIPLEGIC_MYOPATHY_MODEL_UP		695	0.36	0.95
212	GSE18467_LISSENCEPHALIC_VS_GYRENCEPHALIC_CEREBRAL_CORTEX_DN		760	0.36	0.95
213	GSE30874_RESVERATROL-SUPPLEMENTED_VS_UNSUPPLEMENTED_ATHEROGENIC_DIET_4MONTHS_PBMC_UP		480	0.36	0.95
214	GSE33246_HIGH_DOSE_VS_UNTREATED_DON-TREATED_BASOLATERAL_IPEC-J2_UP		765	0.36	0.94
215	GSE22487_D21_VS_D0_NEONATAL_SKELETAL_MUSCLE_DN		650	0.36	0.94

216	GSE28003_STA-KO_STB-LOW_LT-KO_VS_WT_E_COLI_JEJUNUM_4HPI_DN		665	0.36	0.94
217	GSE24239_D5_VS_D1_WASTING-SENSITIVE_SKELETAL_MUSCLE_ACUTE_QUADRIPLEGIC_MYOPATHY_MODEL_DN		705	0.35	0.94
218	GSE73088_LETHAL_DOSE_MUSTARD_GAS_VS_CTRL_LUNG_TISSUE_DN		680	0.36	0.94
219	GSE15256_ANTIBIOTIC-TREATED_ISOLATOR-HOUSED_VS_INDOOR-HOUSED_NEONATAL_JEJUNUM_DN		580	0.36	0.94
220	GSE21071_CYSTIC_FIBROSIS_VS_HEALTHY_NEONATAL_BRONCHUS_UP		680	0.35	0.94
221	GSE65008_SUCKLING_VS_WEANED_35DPN_ILEAL_MUCOSA_DN		755	0.35	0.93
222	GSE34569_INFARCT_REGION_30D_VS_CTRL_MYOCARDIAL_INFARCTION_DN		705	0.35	0.93
223	GSE48839_P2_VS_P0_AORTIC_VALVE_INTERSTITIAL_CELLS_DN		685	0.35	0.93
224	GSE22487_D7_VS_D0_NEONATAL_SKELETAL_MUSCLE_DN		670	0.35	0.93
225	GSE66317_LOW_LACTOFERRIN-SUPPLEMENTED_VS_STANDARD_DIET_JUVENILE_HIPPOCAMPUS_DN		380	0.35	0.92
226	GSE24239_WASTING-SENSITIVE_VS_WASTING-INSENSITIVE_SKELETAL_MUSCLE_ACUTE_QUADRIPLEGIC_MYOPATHY_MODEL_DN		770	0.35	0.92
227	GSE31191_LOW_VS_ADEQUATE_PROTEIN_DIET_YOUNG_LIVER_TISSUE_UP		800	0.35	0.92
228	GSE14790_SUBCLINICAL_PCV2_VS_UNINFECTED_BLOOD_21DPI_DN		715	0.34	0.91
229	GSE28003_WT_VS_CTRL_E_COLI_JEJUNUM_4HPI_UP		740	0.34	0.91
230	GSE16348_D5_VS_D1_IMMOBILIZED_MUSCLE_DN		770	0.34	0.91
231	GSE30956_2H_VS_0H_LPS_STIMULATION_BONE-MORROW_DERIVED_MACROPHAGES_UP		725	0.34	0.91
232	GSE18359_HIGH_VS_LOW_FEED_EFFICACY_AD_LIB_ADIPOSE_TISSUE_UP		595	0.34	0.90
233	GSE72025_SSEA1-NEG_VS_SSEA1-POS_FETAL_FIBROBLASTS_UP		805	0.34	0.90
234	GSE21096_ISCHEMIC_PRECONDITIONED_VS_REPETITIVE_CORONARY_OCCLUSION_MYOCARDIAL_INFARCTION_DN		730	0.34	0.90
235	GSE37166_CORTICOSTEROID-TREATED_VS_UNTREATED_SKELETAL_MUSCLE_ACUTE_QUADRIPLEGIC_MYOPATHY_MODEL_UP		650	0.34	0.90
236	GSE19975_WHITE_VS_RED_SKELETAL_MUSCLE_UP		810	0.34	0.89
237	GSE22596_D2_VS_D0_JEJUNUM_SALMONELLA_TYPHIMURIUM_INFECTION_UP		735	0.34	0.89
238	GSE65008_SUCKLING_VS_WEANED_28DPN_ILEAL_MUCOSA_DN		705	0.33	0.88
239	GSE26663_SEDENTARY_VS_EXERCISE-TRAINED_INTERNAL_MAMMARY_ARTERY_ENDOTHELIUM_DN		730	0.33	0.88

240	GSE18359_AD_LIB_VS_CALORIE-RESTRICTED_LOW_FEED_EFFICACY_LIVER_DN		750	0.33	0.87
241	GSE30956_7H_VS_0H_LPS_STIMULATION_BONE-MORROW_DERIVED_MACROPHAGES_DN		685	0.33	0.87
242	GSE14790_SUBCLINICAL_PCV2_VS_UNINFECTED_BLOOD_7DPI_UP		625	0.33	0.87
243	GSE15256_ANTIBIOTIC-TREATED_ISOLATOR-HOUSED_VS_OUTDOOR-HOUSED_WEANING-AGE_JEJUNUM_UP		600	0.33	0.86
244	GSE22596_D1_VS_D0_ILEUM_SALMONELLA_TYPHIMURIUM_INFECTION_DN		810	0.33	0.86
245	GSE64246_INACTIVATED_S_AUREUS-TREATED_24H_VS_UNTREATED_MAMMARY_EPITHELIAL_CELLS_UP		840	0.32	0.85
246	GSE64246_INACTIVATED_E_COLI-TREATED_24H_VS_UNTREATED_MAMMARY_EPITHELIAL_CELLS_UP		800	0.32	0.84
247	GSE18854_D1_OF_DEDIFFERENTIATION_VS_D0_FOLLICULAR_GRANULOSA_CELLS_UP		690	0.32	0.84
248	GSE15211_NORMOTENSIVE_VS_HYPERTENSIVE_AORTIC_VALVE_INTERSTITIAL_CELLS_UP		775	0.31	0.83
249	GSE33037_ENDOTOXIN-TREATED_VS_UNTREATED_SKELETAL_MUSCLE_ACUTE_QUADRIPLEGIC_MYOPATHY_MODEL_DN		675	0.31	0.82
250	GSE33246_HIGH_DOSE_VS_UNTREATED_DON-TREATED_APICAL_IPEC-J2_UP		670	0.31	0.82
251	GSE24889_STREPTOCOCCUS_SUIS-INFECTED_VS_CTRL_24HPI_BRAIN_TISSUE_UP		445	0.31	0.82
252	GSE22596_D6_VS_D0_COLON_SALMONELLA_TYPHIMURIUM_INFECTION_DN		780	0.31	0.82
253	GSE41636_LEAN_VS_OBESE_CORONARY_ARTERY_UP		710	0.30	0.80
254	GSE48839_TGF1BETA-TREATED_VS_UNTREATED_24H_AORTIC_VALVE_INTERSTITIAL_CELLS_DN		795	0.30	0.80
255	GSE24889_STREPTOCOCCUS_SUIS-INFECTED_VS_CTRL_24HPI_PBMC_DN		520	0.30	0.80
256	GSE22596_D6_VS_D0_ILEUM_SALMONELLA_TYPHIMURIUM_INFECTION_UP		860	0.29	0.76
