# Supplementary material for: Identification of the genetic basis of sow pelvic organ prolapse
Source: Front Genet. 2023 Apr 18;14:1154713. doi: 10.3389/fgene.2023.1154713 (PMC10151575; doi:10.3389/fgene.2023.1154713)
Supplement: Supplementary file 1 [file DataSheet1.zip › Supplemental_Material/Image 1.pdf]

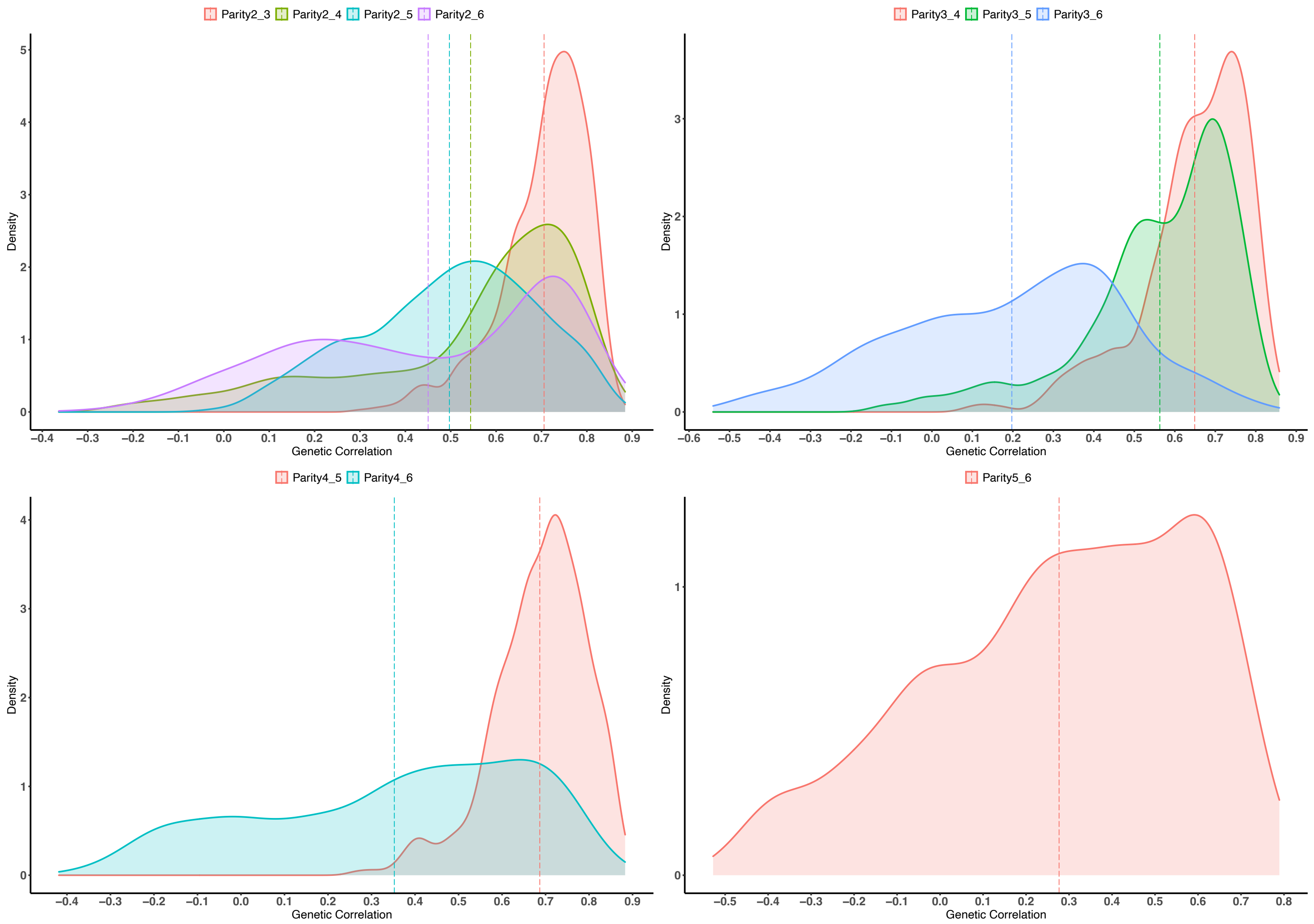

Figure S1. Posterior distributions of genetic correlations for pelvic organ prolapse (POP) phenotypes between parities based on bivariate Bayes-C0 analyses.

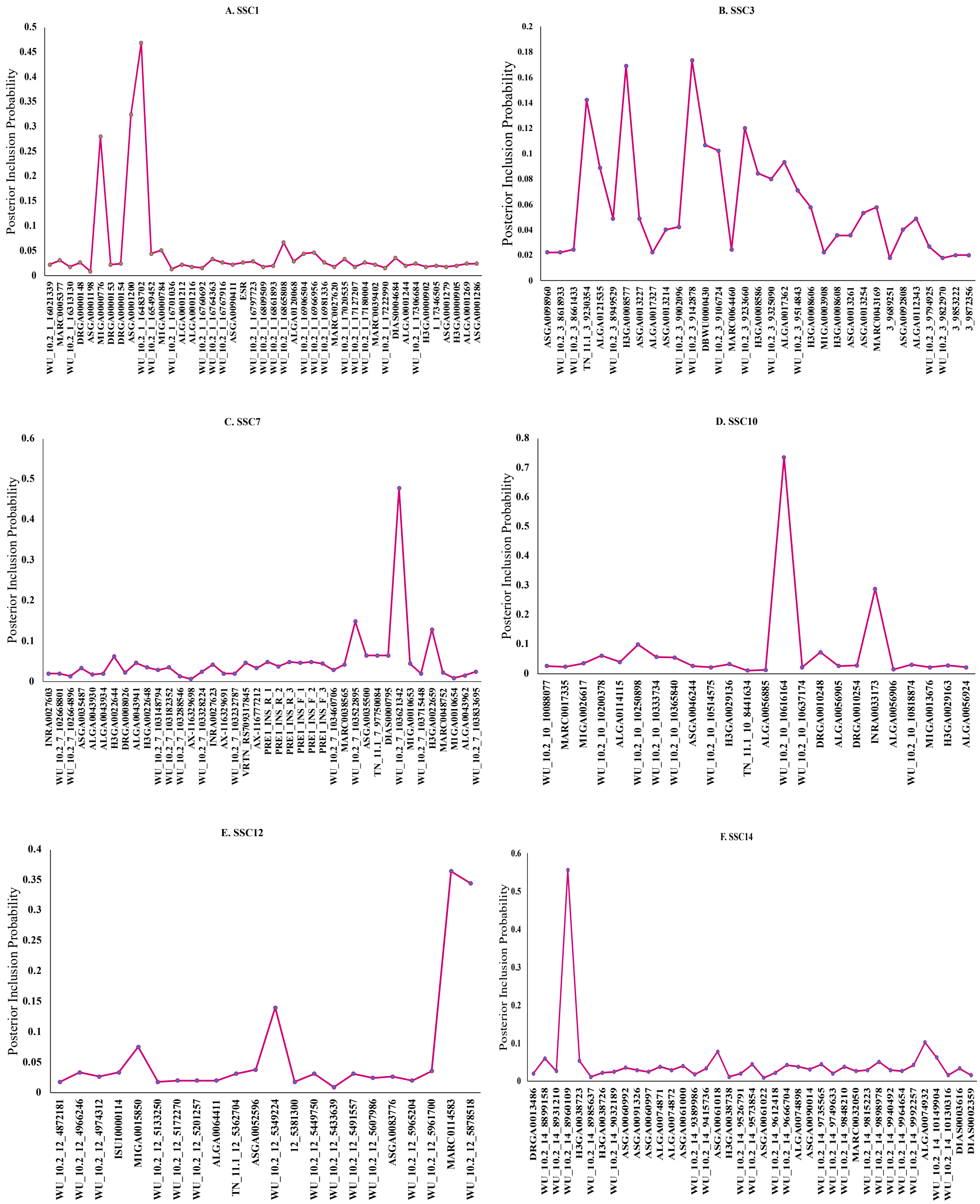

Figure S2. Posterior Inclusion Probability of markers included in the non-overlapping 1 Mb window across the genome that explained >1% of the genetic variance in the across-parity analysis, as identified in Table 2.
